# Optimization Method for Robustness of Hypernetwork Communication with Integrated Structural Features

**DOI:** 10.3390/e28010075

**Published:** 2026-01-09

**Authors:** Lei Chen, Xiujuan Ma, Fuxiang Ma

**Affiliations:** 1 School of Computer Science, Qinghai Normal University, Xining 810008, China; 202533341006@stu.qhnu.edu.cn (L.C.);; 2 The State Key Laboratory of Tibetan Intelligence, Qinghai Normal University, Xining 810008, China

**Keywords:** hypernetwork, communication robustness, hyper-betweenness centrality, hyper-centrality of feature subgraph, fiedler, hyperdistance entropy

## Abstract

The ultimate objective of research on hypernetwork robustness is to enhance its capability to withstand external attacks and natural disasters. For hypernetworks such as telecommunication networks, public safety networks, and military networks—where security requirements are extremely high—achieving higher communication robustness is essential. This study integrates the structural characteristics of hypernetworks with an optimization method for communication robustness by combining four key indicators: hyper-betweenness centrality, hyper-centrality of feature subgraph, hyper-centrality of Fiedler, and hyperdistance entropy. Using the best improvement performance (BIP_T) as the evaluation metric, simulation experiments were conducted to comparatively analyze the effectiveness of these four indicators in enhancing the communication robustness of Barabási–Albert (BA), Erdos–Renyi (ER), and Newman–Watts (NW) hypernetworks, and theoretically derives the hyperedge addition threshold θ. The results show that all four indicators effectively improve the communication robustness of hypernetworks, although with varying degrees of optimization. Among them, hyper-betweenness centrality demonstrates the most significant optimization effect, followed by hyper-centrality of feature subgraph and hyper-centrality of Fiedler, while hyperdistance entropy exhibits a relatively weaker effect. Furthermore, these four indicators and the proposed communication robustness optimization method exhibit strong generalizability and have been effectively applied to the WIKI-VOTE social hypernetwork.

## 1. Introduction

The emergence of multidimensional interactions has made it increasingly difficult for complex networks based on binary relationships to accurately represent multidimensional complex systems. For example, social networks based on ordinary graphs can only represent the interactions between any two individuals and are unable to capture the multidimensional interaction relationships among multiple individuals. Existing studies have shown that hypernetworks, based on hypergraph theory, have become a powerful tool for representing multidimensional complex systems [1]. Currently, hypernetworks have attracted widespread research, covering areas such as model construction [2,3,4,5], topology structure mining [6,7,8], control [9,10], and information propagation analysis [11,12,13]. These efforts have led to many important research outcomes, which have laid a solid theoretical foundation for the in-depth analysis of multidimensional complex systems.

Robustness, as a measure of a network’s ability to maintain stable operation under disturbances, is an important indicator of its resistance to disruptions, such as physical failures, natural disasters, and malicious attacks [14]. In recent years, robustness has garnered significant attention from researchers across various network fields, especially in networks with high security requirements, such as military networks [15], public safety networks [16,17], and others. In these networks, every communication opportunity is crucial for users. Therefore, under various disruptive conditions, how to effectively enhance the communication robustness of the network is a key research topic.

In recent years, researchers have made significant progress in enhancing the communication robustness of the networks [18,19,20]. The literature [21] provides a systematic review of the theoretical modeling and computational methods for complex networks in response to perturbations. However, most of the studies mentioned focus on complex networks based on binary relationships, which limits their ability to characterize higher-order interactions in hypernetworks. To address this, Chen et al. [22] proposed two methods to optimize the connectivity robustness of hypernetwork structures: random hyperedge recombination and preferential hyperedge recombination of low-degree nodes. However, these methods primarily focus on improving the global connectivity of the hypernetwork and fail to fully consider the communication reliability between node pairs, making them difficult to directly apply to scenarios where the goal is to optimize communication robustness between nodes. Zhang et al. [23] focused on evaluating the robustness of cell–cell communication inference methods under different data perturbations. This study emphasizes the quantitative evaluation and comparison of methodological robustness, without addressing optimization from the perspective of network structure. Lin et al. [24] used personalized federated learning to reconcile the differences between global and local models in low-dimensional subspaces, thereby balancing communication efficiency, robustness, and fairness. However, the robustness improvement of this method primarily relies on optimization at the model parameter level, with less attention paid to the topology of the communication network itself. Nie et al. [25] proposed a multi-objective robustness optimization algorithm based on a weighted model to enhance the robustness of complex networks based on binary interactions. However, this algorithm is not applicable to hypernetwork structures based on multidimensional interactions. These studies show that, despite the extensive work done on improving network robustness, existing methods often focus on single-level optimizations and fail to fully consider the complex multidimensional interactions in hypernetworks and the communication robustness between nodes.

To enhance the communication robustness of hypernetworks under node failures, this paper proposes a novel algorithmic optimization method based on four hypernetwork topological parameters: hyper-betweenness centrality, hyperdistance entropy, the hyper-centrality of feature subgraphs, and the Fiedler value. The proposed method improves communication robustness through structural optimization by strategically adding hyperedges to the hypernetwork. Specifically, we evaluate the optimization performance of the proposed method on three representative synthetic hypernetworks: the Barabási–Albert (BA) hypernetwork, the Erdős–Rényi (ER) hypernetwork, and the Newman–Watts (NW) hypernetwork. In addition, we analyze how hypernetwork structural parameters and different attack strategies influence the effectiveness of communication robustness optimization. Furthermore, using social relationship data from WIKI-VOTE, we construct a real-world social hypernetwork and conduct empirical analyses to further demonstrate the applicability and effectiveness of the proposed optimization method.

## 2. Related Knowledge

### 2.1. Uniform Hypergraph and Non-Uniform Hypergraph

**Figure 1:**
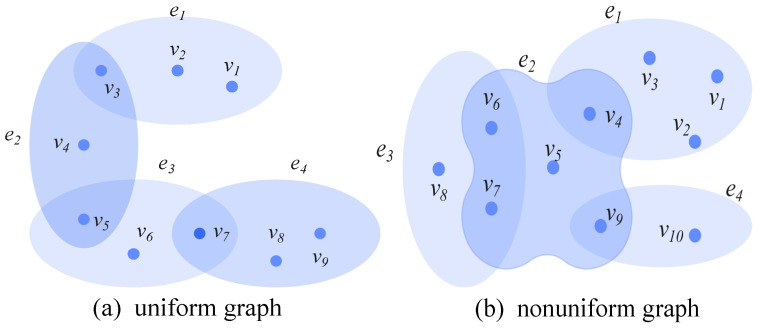
Hypergraph.

To further analyze the applicability of the optimization method under different structures, this paper studies the Communication robustness of both uniform and non-uniform hypergraphs. Let the hypergraph H=(V,E) denote a hypergraph, where N(H) represents the number of nodes in *H*, and M(H) denotes the size of the hyperedges, i.e., the number of hyperedges [26]. Let $|e_{i}|$ denote the number of nodes contained in the hyperedge $e_{i}$. The rank of the hypergraph *H* is defined as the maximum number of nodes contained in a hyperedge, denoted as $r\left(H\right)=\max_{i}\left|e_{i}\right|$, where (i=1,2,⋯,M). The co-rank of the hypergraph *H* refers to the minimum number of nodes contained in a hyperedge, denoted as $cr\left(H\right)=\min_{i}\left|e_{i}\right|$, where (i=1,2,⋯,M). If r(H)=cr(H)=k, the hypergraph *H* is called a *k*-uniform hypergraph or a uniform hypergraph [26]. Conversely, if r(H)≠cr(H), the hypergraph *H* is termed a non-uniform hypergraph. Figure 1a shows a 3-uniform hypergraph with 9 nodes and 4 hyperedges, while Figure 1b shows a non-uniform hypergraph with 10 nodes and 4 hyperedges.

### 2.2. Hypernetwork Communication Robustness Evaluation Metrics

To effectively enhance the communication robustness of hypernetworks, selecting appropriate node positions for adding hyperedges is crucial. For this purpose, we use four key metrics to assess the position and importance of nodes: hyper-betweenness centrality (HBC), hyper-centrality of feature subgraph (HC_sub), hyper-centrality of Fiedler (HC_Fiedler), and hyperdistance entropy (HDI). The following will elaborate on these four indicators in detail.

#### 2.2.1. Hyper-Betweenness Centrality

In hypernetwork analysis, the role of nodes on the shortest hyperpaths has a decisive impact on overall communication efficiency. Hyper-betweenness centrality (HBC) [27] measures the extent to which a node acts as a “bridge” or “bottleneck” in the shortest hyperpaths, and is therefore the most direct indicator for characterizing a node’s contribution to communication connectivity. From the perspective of communication robustness, the failure of a node on a critical path can significantly reduce information transmission efficiency and may even lead to local disconnections, whereas enhancing the structural redundancy of such nodes typically improves the network’s resistance to attacks. Thus, introducing the HBC metric not only helps identify nodes crucial to communication paths but also provides a theoretical foundation for further improving the global communication robustness of hypernetworks through hyperedge augmentation. The definition of hyper-betweenness centrality (HBC) is given by Equation (1):

(1) $$HBC\left(\nu_{i}\right)=\frac{2}{\left(N-1)(N-2\right)}\sum_{\begin{array}{c} j,k\in V\\ j\neq i,k\neq i,j<k \end{array}}\frac{n_{jk}\left(\nu_{i}\right)}{n_{jk}}.$$

where $n_{jk}$ represents the number of shortest hyperpaths [26] connecting nodes *j* and *k*; $n_{jk}\left(v_{i}\right)$ denotes the number of shortest hyperpaths connecting nodes *j* and *k* that pass through node *i*. The higher the value of a node’s hyper-betweenness centrality, the more shortest paths pass through that node, indicating its central position in the network. Conversely, the lower the value, the more peripheral the node is within the network.

#### 2.2.2. Hyper-Centrality of Feature Subgraph

The local structural characteristics in hypernetworks play a crucial role in enhancing their communication robustness. Hypercharacteristic Subgraph Centrality (HC_sub) [28], derived from spectral graph theory, measures the degree of a node’s participation in the local structure by analyzing the characteristic information of the adjacency matrix. Existing studies have shown that the stability of local structures can effectively inhibit the rapid spread of noise or local perturbations in the network, thereby enhancing the network’s robustness when facing small-scale failures or perturbations. Therefore, the introduction of the HC_sub index helps identify nodes that play key roles in the local structure. Strengthening these nodes can improve the local robustness of the hypernetwork, providing support for enhancing the overall communication robustness. The definition of hyper-centrality of feature subgraph is given by Equation (2):

(2) $$HC\_sub\left(e_{a}\right)=\sum_{i=1}^{N}{\left(u_{i}\left(e_{a}\right)\right)}^{2}e^{\lambda_{i}}.$$

When calculating the hyper-centrality of feature subgraph for nodes in a hypernetwork, the hyper-centrality of feature subgraph for each hyperedge must first be computed. Then, based on the weights of the nodes within the hyperedge, the hyper-centrality of feature subgraph for the hyperedge is proportionally distributed among the nodes within that hyperedge. The specific calculation steps are as follows:

(1) Convert the hypernetwork into a line graph and calculate the hyper-centrality of feature subgraph for each hyperedge $HC\_sub\left(e_{a}\right)=\sum_{i=1}^{N}{\left[u_{i}\left(e_{a}\right)\right]}^{2}e^{\lambda_{i}}$ based on the hyperedge adjacency matrix. Here, $\lambda_{i}$ denotes the *i*-th eigenvalue of the hyperedge adjacency matrix, and $u_{i}\left(e_{a}\right)$ represents the value of feature vector $u_{i}$ corresponding to feature value $\lambda_{i}$ at node *i*.

(2) Select one hyperedge $e_{a}$ and identify all the nodes contained in hyperedge $e_{a}$, forming a node set $V_{e_{a}}$. Assign a weight *w* to each node in the node set, where the weight of node *n* is given by Equation (3):

(3) $$w_{n}=\frac{\mathrm{Hyperdegree}(n)}{\sum_{m\in V_{e_{a}}}\mathrm{Hyperdegree}\left(m\right)}.$$

(3) Calculate the hyper-centrality of feature subgraph for each node in the hyperedge, $HC\_sub\left(v_{e_{a}}\right)=w_{n}\times HC\_sub\left(e_{a}\right)$.

(4) Identify the hyperedges that each node *v* belongs to, forming the set of hyperedges $E_{v}$ that contain node *v*. Calculate the hyper-centrality of feature subgraph $HC\_sub\left(v\right)=\sum_{a}^{E_{v}}HC\_sub\left(v_{e_{a}}\right)$ for each node in the hypernetwork.

The calculation results show that the larger the value of a node’s hyper-centrality of feature subgraph in the hypernetwork, the more central the node is within the network. Conversely, the smaller the value, the more peripheral the node is in the network.

#### 2.2.3. Hyper-Centrality of Fiedler

The overall connectivity of a hypernetwork is a core indicator for measuring its communication robustness, and the Fiedler value in spectral graph theory [29] is considered a key quantity for describing structural stability and global connectivity. Based on this, this paper introduces the hyper-centrality of Fiedler (HC_Fiedler), which identifies key nodes that maintain the overall structural stability by calculating the degree of influence of nodes on the algebraic connectivity of the hypernetwork. The failure of such nodes often leads to a significant decrease in the hypernetwork’s connectivity, thereby weakening the network’s resistance to perturbations. Therefore, by using the HC_Fiedler metric to screen key nodes and structurally enhance them, the communication robustness of the hypernetwork in a global sense can be effectively improved. The definition of the Fiedler centrality (HC_Fiedler) is given by Equation (4):

(4) $$HC\_Fiedler\left(v_{i}\right)=\sum_{j=1}^{\left|\Gamma (v_{i})\right|}\left|R_{\mathrm{Fiedler}}\left(v_{i}\right)-R_{\mathrm{Fiedler}}\left(v_{j}\right)\right|.$$

When calculating the Fiedler centrality of nodes in a hypernetwork, the Fiedler value for each hyperedge must first be computed. Then, based on the weights of the nodes within the hyperedge, the Fiedler value of the hyperedge is proportionally distributed among the nodes in that hyperedge. The specific calculation steps are as follows:

(1) Convert the hypernetwork into a line graph, and calculate the Fiedler value for each hyperedge based on the Laplacian matrix of the line graph: $HC\_Fiedler\left(e_{a}\right)=\sum_{j=1}^{\left|\Gamma (e_{a})\right|}\left|R_{\mathrm{Fiedler}}\left(e_{a}\right)-R_{\mathrm{Fiedler}}\left(e_{\tau_{j}}\right)\right|$, where, $\Gamma (e_{a})$ represents the set of nodes connected to hyperedge $e_{a}$ in the line graph. After identifying the Laplacian matrix of the line graph, the second smallest eigenvalue of this Laplacian matrix, which is the Fiedler value, can be calculated. Next, the corresponding eigenvector $u_{\mathrm{Fiedler}}$ for the Fiedler value is identified. $R_{\mathrm{Fiedler}}\left(e_{a}\right)$ represents the $e_{a}$-th element of the eigenvector $u_{\mathrm{Fiedler}}$.

(2) Select one hyperedge $e_{a}$ and identify all the nodes contained in hyperedge $e_{a}$, forming the node set $V_{e_{a}}$. Assign a weight *w* to each node in the node set, where the weight of node *n* is given by Equation (3).

(3) Calculate the hyper-centrality of the feature subgraph for each node in hyperedge $e_{a}$: $HC\_Fiedler\left(v_{e_{a}}\right)=w_{n}\times HC\_Fiedler\left(e_{a}\right)$.

(4) Identify the hyperedges that each node *v* belongs to, forming the set of hyperedges $E_{v}$ that contain node *v*. Calculate the hyper-centrality of feature subgraph for each node in the hypernetwork: $HC\_Fiedler\left(v\right)=\sum_{a\in E_{v}}HC\_Fiedler\left(v_{e_{a}}\right)$.

The calculation results show that the larger the Fiedler value of a node in the hypernetwork, the more central the node is within the network. Conversely, the smaller the Fiedler value, the more peripheral the node is in the network.

#### 2.2.4. Hyperdistance Entropy

Communication robustness is not only affected by path structures or local factors, but also closely related to the distance distribution of nodes in the hypernetwork. Hyperdistance entropy (HDI) [30] reflects the “centrality” of a node and the efficiency of information diffusion by measuring the dispersion degree of the shortest hyperpath lengths from the node to other nodes. Nodes with uniform distance distribution and small average distance are usually located at the communication center of the hypernetwork, which play a crucial role in improving the overall communication efficiency and reducing the delay caused by failures. In contrast, nodes with large distance entropy tend to be at the edge of the hypernetwork and are potential vulnerable parts of the hypernetwork. Therefore, the introduction of the HDI index can identify edge nodes or central nodes that should be focused on in robustness optimization from the perspective of distance structure, providing a theoretical basis for improving the overall communication performance of the hypernetwork. The definition of the hyperdistance entropy (HDI) in the hypernetwork is given by Equation (5):

(5) $$HDI\left(v_{i}\right)=-\sum_{j=1}^{d_{\max}}P_{j}\log P_{j}.$$

where *N* is the total number of nodes in the hypernetwork; $P_{j}$ represents the ratio of the number of nodes at a hyperpath distance of length *j* from node *i* to N−1; $d_{\max}=\sup\left\{d\left(v_{i},u\right)|u\in V\right\}$. The smaller the value of a node’s distance entropy, the more central the node is within the hypernetwork. Conversely, the larger the value of the distance entropy, the more peripheral the node is in the hypernetwork.

The above four types of indicators reveal the mechanism of nodes’ role in robustness from different dimensions such as path structure, local structure, global connectivity, and distance distribution, providing a more comprehensive and reliable structural basis for the optimization of communication robustness.

### 2.3. Calculation Example

**Figure 2:**
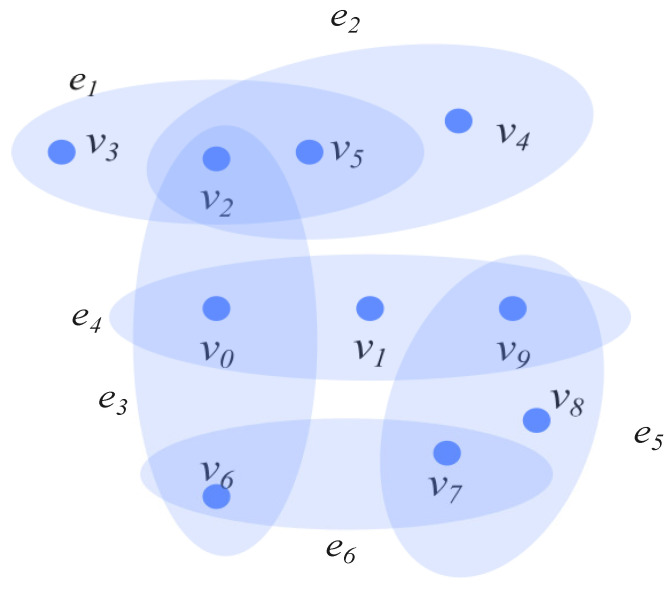
Example hypergraph.

In order to more intuitively compare the similarities and differences between the four metrics, this section analyzes the hypernetwork *H*. As shown in Figure 2, this hypernetwork contains 10 nodes and 6 hyperedges.

By calculation, the hyper-betweenness centrality, hyperdistance entropy, hyper-centrality of feature subgraph, and Fiedler-based centrality values for each node in hypernetwork *H* are obtained. The nodes are then ranked according to their positions to reveal the similarities and differences in how the various metrics assess node importance. The higher the rank of a node, the closer its position is to the center of hypernetwork *H*; conversely, the lower the rank, the more peripheral the node is in the hypernetwork. The ranking of nodes in hypernetwork *H* based on the four centrality metrics is shown in Table 1:

## 3. Optimization Method Based on Communication Robustness Metrics

**Figure 3:**
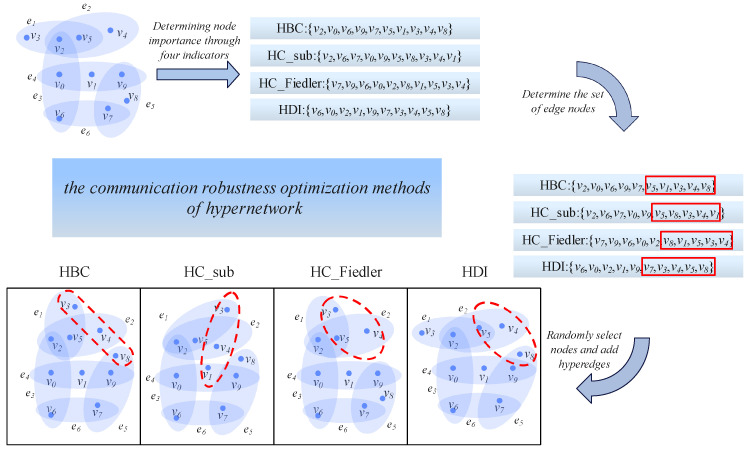
Method flowchart. Herein, the nodes marked by red boxes are those contained in the newly added hyperedges, and the red circles represent the newly added hyperedges in the hypernetwork.

The optimization method for communication robustness in hypernetworks is based on hyper-betweenness centrality, hyperdistance entropy, hyper-centrality of feature subgraph, and Fiedler-based centrality. This method identifies the set of peripheral nodes in the hypernetwork, and different nodes are selected from this set to add hyperedges, thereby improving the communication robustness of the hypernetwork. The process of the optimization method for communication robustness is shown in Figure 3:

Based on the methodological framework illustrated in Figure 3, the specific implementation procedure of the proposed optimization method is described as follows.

First, given an initial hypernetwork $H_{0}$, we compute four centrality metrics for each node: hyperdegree-based centrality (HBC), distance-entropy centrality (HDI), subgraph centrality (HC_sub), and Fiedler-eigenvalue-based centrality (HC_Fiedler). These metrics are evaluated to identify peripheral nodes in the network from multiple perspectives. For each centrality measure, nodes with lower rankings (i.e., smaller centrality values), such as those highlighted by the red boxes in Figure 3, are selected to form the corresponding candidate set of peripheral nodes.

Next, the number of nodes contained in each newly added hyperedge, denoted as add_node, is determined. If $H_{0}$ is a uniform hypernetwork in which all hyperedges contain *k* nodes, then add_node = k. Otherwise, we compute the average hyperedge cardinality *m* of the network and set add_node = m. This design ensures that the size of newly added hyperedges remains compatible with the connectivity pattern of the original network.

Subsequently, according to the selected optimization strategy (Method), add_node nodes are randomly sampled from the corresponding peripheral-node candidate set to form a new hyperedge. This “selection–addition” process is repeated until the number of added hyperedges reaches the predefined addition ratio *q*, defined as the proportion of newly added hyperedges relative to the initial total number of hyperedges *M*. The optimized hypernetwork obtained after this process is denoted as $H_{q}$.

To evaluate the effectiveness of the optimization, we compare the communication efficiency of the original hypernetwork $H_{0}$ and the optimized hypernetwork $H_{q}$ under the same attack strategy. The primary evaluation metric is the best improvement performance (BIP_T), which quantifies the maximum relative improvement in communication efficiency achieved by the optimized network over the original one at a given attack intensity. The complete procedure of the proposed method is summarized in Algorithm 1:
**Algorithm 1:** Robustness optimization method for communication in hypernetworks**Step****Description**inputInitial Hypernetwork $H_{0}$, Total hyperedge number *M*, Proportion of newly added hyperedges *q*, Communication Robustness Optimization Method MethodoutputCommunication Efficiency of $H_{0}$, BIP_TInitializationgenerating hypernetwork $H_{0}$ with *M* hyperedges1do Calculate for each node in $H_{0}$, its hyper-betweenness, distance entropy, characteristic subgraph centrality, and Fiedler-value-based centrality, and, for each of these centrality measures, identify the corresponding set of peripheral nodes $V_{\mathrm{margin}\_\mathrm{HBC}}$, $V_{\mathrm{margin}\_\mathrm{HDI}}$, $V_{\mathrm{margin}\_\mathrm{HC}\_\mathrm{sub}}$, and $V_{\mathrm{margin}\_\mathrm{HC}\_\mathrm{Fiedler}}$.2if $H_{0}$ is *k*-uniform hypernetwork:3   do add_node = *k*4else: do Calculate the average number *m* of nodes contained in the hyperedges of the hypernetwork;5   do add_node = *m*6switch(Method) {7   case HBC:8      do from the peripheral node set $V_{\mathrm{margin}\_\mathrm{HBC}}$, select add_node nodes to form a new hyperedge, and repeat this edge-addition process until the prescribed edge-addition ratio is reached;9      break;10   case HDI:11      do from the peripheral node set $V_{\mathrm{margin}\_\mathrm{HDI}}$, select add_node nodes to form a new hyperedge, and repeat this edge-addition process until the prescribed edge-addition ratio is reached;12      break;13   case HC_sub:14      do from the peripheral node set $V_{\mathrm{margin}\_\mathrm{HC}\_\mathrm{sub}}$, select add_node nodes to form a new hyperedge, and repeat this edge-addition process until the prescribed edge-addition ratio is reached;15      break;16   case HC_Fiedler:17      do from the peripheral node set $V_{\mathrm{margin}\_\mathrm{HC}\_\mathrm{Fiedler}}$, select add_node nodes to form a new hyperedge, and repeat this edge-addition process until the prescribed edge-addition ratio is reached;18      break;}19do Denote the hypernetwork with the newly added hyperedges as $H_{q}$;20do Independently attack the hypernetworks $H_{0}$ and $H_{q}$, and compute the communication efficiency CE of both hypernetworks.21do Calculate BIP_T

## 4. Simulation Experiment and Result Analysis

### 4.1. Simulation Experiments and Parameter Settings

To validate the effectiveness of the hypernetwork communication robustness optimization methods proposed in this paper, the following experiments were designed in this paper. Based on the three classic hypernetworks (Barabási–Albert, Erdos–Renyi and Newman–Watts), six types of hypernetworks were constructed, namely k-uniform Barabási–Albert hypernetwork (UBA), non-uniform Barabási–Albert hypernetwork (NUBA), k-uniform Erdos–Renyi hypernetwork (UER), non-uniform Erdos–Renyi hypernetwork (NUER), k-uniform Newman–Watts hypernetwork (UNW), and non-uniform Newman–Watts hypernetwork (NUNW). In hypernetwork robustness research, random attacks(RA) and targeted attacks(TA) are widely used to evaluate the network’s resilience to attacks. In this paper, both random attacks and targeted attacks are employed to verify the effectiveness of the four metrics in enhancing the communication robustness of the six hypernetworks under the hypernetwork communication robustness optimization method. A random attack involves randomly selecting nodes in the network for attack, while a targeted attack prioritizes attacking nodes with a higher hyperdegree in the hypernetwork. The experimental parameters are shown in Table 2. To ensure the accuracy of the experimental results, all results are averaged over 50 experiments.

In many complex networks, communication efficiency is closely related to the network’s robustness [31]. Similarly, this is also the case in hypernetworks. The higher the communication efficiency of a hypernetwork, the smaller the distance between any two nodes in the hypernetwork, meaning that the connection between any two nodes is tighter, which indicates a higher communication robustness of the hypernetwork. Using communication efficiency to measure hypernetwork robustness has been applied in several research studies [21]. The specific definition of communication efficiency in hypernetworks is given by Equation (6):

(6) $$CE=\frac{1}{N}\sum_{i=0}^{N-1}\sum_{j=1}^{\Gamma (i)}\frac{S_{j}^{2}}{N^{2}}.$$

where, *N* represents the number of nodes in the hypernetwork, Γ(i) denotes the number of connected components in the network after the *i*-th node is attacked (where *i* can take values up to N−1), and $S_{j}$ represents the number of nodes in the *j*-th connected component.

In order to better assess the changes in the hypernetwork structure brought about by the proposed hypernetwork communication robustness optimization method and to quantitatively evaluate the optimization effect of this method, this section uses the evaluation metric BIP_T (Best Improvement Performance-Transmission) to represent the rate of change in communication efficiency. Specifically, BIP_T is used to measure the impact of the hypernetwork connectivity robustness optimization method on hypernetwork communication robustness. The specific definition is given by Equation (7):

(7) $$BIP\_T=\frac{\Phi \left(H,OM\right)-\Phi \left(H_{0}\right)}{\Phi (H_{0})}.$$

where $\Phi (H_{0})$ represents the communication efficiency of the initial hypernetwork $H_{0}$ after being attacked, and Φ(H,OM) represents the communication efficiency of the hypernetwork *H* after being attacked, where the optimization method (OM) has been applied.

### 4.2. Impact of Hyperedge Addition Ratio q on Communication Robustness and Threshold Analysis

**Figure 4:**
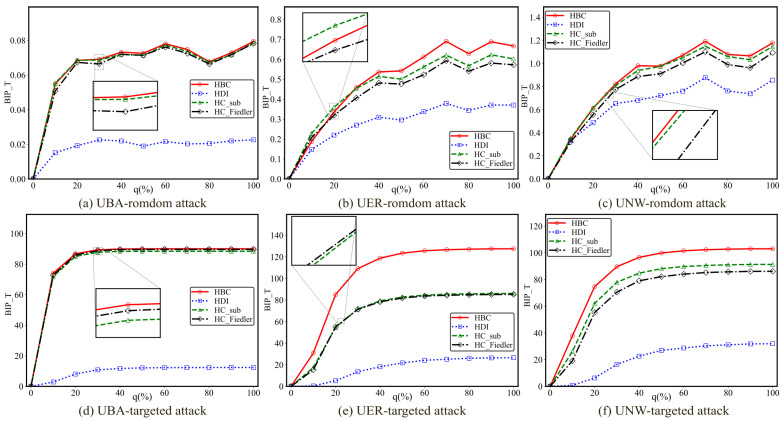
The BIP_T of three types of uniform hypernetworks under different attack methods with varying proportions of added hyperedges q. The total number of hyperedges is *M* = 1500. The variation in the best improvement in communication robustness with respect to the proportion of added hyperedges q is shown for three types of uniform hypernetworks (UBA, UER, and UNW) under random and targeted attacks.

**Figure 5:**
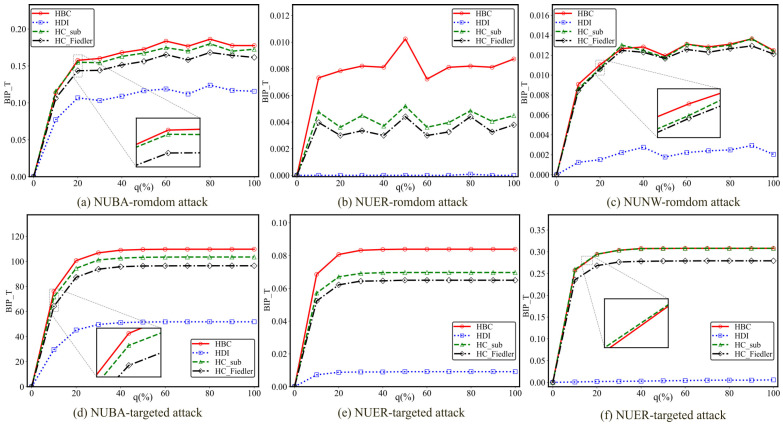
The BIP_T of three types of non-uniform hypernetworks under different attack methods with varying proportions of added hyperedges q. The total number of hyperedges is *M* = 1500. The variation in the best improvement in communication robustness with respect to the proportion of added hyperedges q is shown for three types of non-uniform hypernetworks (NUBA, NUER, and NUNW) under random and targeted attacks.

This subsection conducts comparative experiments on six different hypernetworks to investigate the impact of the newly added hyperedge ratio *q* on the experimental outcomes. To eliminate the influence of network scale differences on the results and to ensure comparability across different values of the added hyperedge ratio, a unified experimental scale of 1500 hyperedges is adopted. This setting not only preserves the structural complexity of the hypernetwork but also balances computational efficiency and experimental stability. The experimental results are presented in Figure 4 and Figure 5, respectively. In each subfigure, the horizontal axis represents the added hyperedge ratio *q*, while the vertical axis denotes the optimal improvement in communication robustness.

As shown in Figure 4, the newly added hyperedge ratio *q* has a significant impact on the experimental results. With the gradual increase of *q*, the communication robustness improvement achieved by all methods generally exhibits a trend of rapid growth followed by a gradual saturation. This indicates that when the hyperedge addition ratio is relatively low, the introduction of new hyperedges can substantially enhance the network connectivity, thereby leading to a rapid improvement in its resilience against attacks. However, once *q* exceeds a certain threshold, the network structure becomes increasingly saturated, and the marginal contribution of additional hyperedges to overall robustness is markedly reduced, causing the best improvement performance to enter a stable phase.

As shown in Figure 5, when *q* is small, the improvement in communication robustness increases rapidly with *q*, suggesting that the introduction of a limited number of critical hyperedges is sufficient to significantly enhance the overall connectivity of the network. As *q* increases beyond a certain range, the improvement curves of all methods gradually flatten and approach a saturation regime. This phenomenon demonstrates that, in non-uniform hypernetworks, the enhancement of communication robustness through hyperedge addition also exhibits diminishing marginal returns, and excessively high hyperedge addition ratios fail to yield proportional performance gains.

Moreover, as the hyperedge addition ratio *q* increases, the optimization methods based on all four metrics exhibit a common pattern characterized by rapid initial improvement followed by convergence to a stable regime, indicating the existence of a saturation point for hyperedge addition. In practical applications, once the number of added hyperedges exceeds this saturation threshold, further additions not only fail to produce additional benefits but may also incur unnecessary costs. To achieve optimal performance while controlling costs in complex systems, we next theoretically derive the hyperedge addition thresholds corresponding to the metric-fusion–based optimization methods under different network structures and attack scenarios.

To characterize the variation trend of the optimal improvement performance of hypernetwork communication robustness with respect to the hyperedge addition ratio, an exponential model is initially adopted as follows:

(8) $$BIP\_T=e^{q},$$

where *q* denotes the hyperedge addition ratio (q≥0). However, the basic exponential function exhibits a monotonically increasing behavior, which is inconsistent with the saturation-type growth pattern observed in the experiments, namely, a rapid increase in the initial stage followed by a gradual stabilization. Therefore, to better fit the experimental data, Equation (8) is modified to obtain Equation (9). Specifically, the function is transformed by performing symmetry operations with respect to both the *y*-axis and the *x*-axis, enabling it to capture the characteristic behavior of rapid initial growth followed by saturation. This transformation aligns with the empirical observation that the optimal improvement performance increases sharply at the early stage of optimization and gradually converges thereafter. Meanwhile, an upward translation by one unit along the *y*-axis is applied to better fit the initial growth trend of the curve. The resulting expression is given in Equation (9).

(9) $$BIP\_T_{1}=1-e^{-q},$$

Since the maximum values of different curves vary, a parameter *a* is introduced to control the upper bound of the function, thereby allowing it to better match the numerical range of the experimental data. The corresponding expression is given in Equation (10).

(10) $$BIP\_T_{2}=a\left(1-e^{-q}\right),$$

Further examination of the experimental results reveals that the growth rates of different curves are not identical. Accordingly, *a* parameter *b* is introduced to adjust the growth speed of the function, enabling a more accurate fit to the varying growth rates of individual curves. The resulting formulation is presented in Equation (11).

(11) $$BIP\_T_{3}=a\times \left(1-e^{-bq}\right),$$

Through the above sequence of transformations, the final expression describing the relationship between the best improvement performance BIP_T and the hyperedge addition ratio *q* is obtained as follows:

(12) $$BIP\_T=a\times (1-e^{-bq}),$$

Considering that most real-world networks exhibit non-uniform topological structures, we focus primarily on the fitting performance in non-uniform hypernetworks. Based on the experimental data described above, we derive the corresponding theoretical fitting functions for BA, ER, and NW hypernetworks with a fixed scale of 1500 hyperedges under different degrees of uniformity and attack scenarios. The fitted theoretical expressions for non-uniform hypernetworks are summarized in Table 3, while the corresponding fitting results for uniform hypernetworks are provided in Table A1 of the Appendix A.

**Figure 6:**
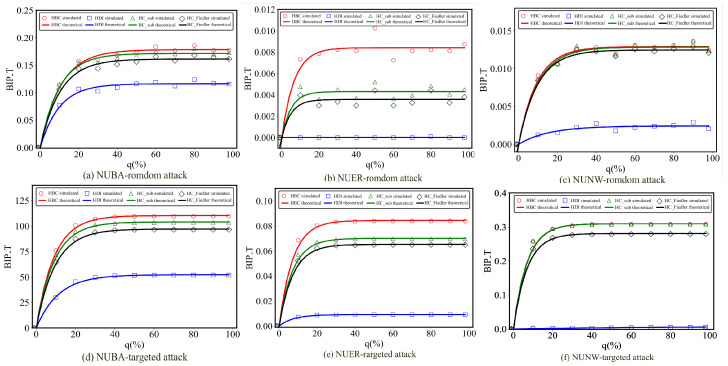
Comparison of theoretical and simulated optimal improvement performance in three types of non-uniform hypernetworks (NUBA, NUER, NUNW) with a total of 1500 hyperedges.

**Figure A1:**
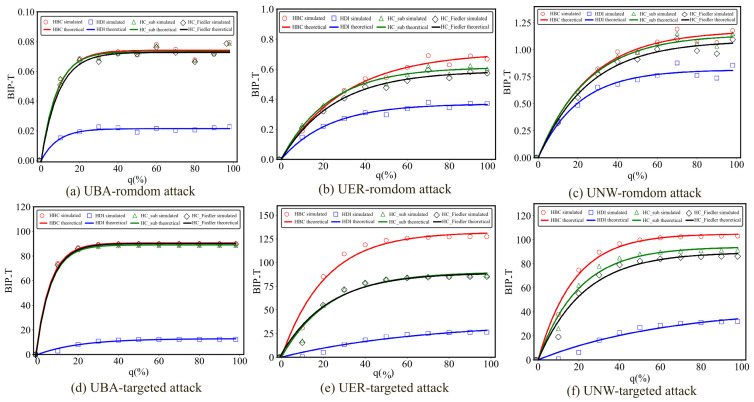
Comparison of theoretical and simulated optimal improvement performance in three types of uniform hypernetworks (UBA, UER, UNW) with a total of 1500 hyperedges.

Through simulation experiments, we obtain the fitting results for all curves corresponding to different hypernetworks. Figure 6 demonstrates the fitting effects of NUBA, NUER and NUNW hypernetworks under different attack strategies. Similarly, the fitting results for uniform hypernetworks are presented in Figure A1 of the Appendix A.

The experimental results demonstrate that, in both uniform and non-uniform hypernetworks, Equation (12) can accurately fit all curves, thereby validating the rationality of determining the hyperedge addition threshold based on Equation (12).

Taking the derivative of Equation (12) yields Equation (13):

(13) $$BIP\_T^{\prime }=a\times b\times e^{-bq},$$

(14) $$a\times b\times e^{-bq}<\varepsilon ,$$

Equation (14) is only valid after *q* reaches a specific threshold, when *q* is smaller threshold, the growth rate of the BIP_T curve remains large until it reaches the flat region. Equation (14) can be simplified to obtain Equation (15):

(15) $$e^{-bq}<\frac{\varepsilon }{a\times b},$$

Taking the logarithm of both sides yields Equation (16):

(16) $$-bq<\ln\frac{\varepsilon }{a\times b},$$

where b>0, further simplification leads to Equation (17):

(17) $$q>-\frac{1}{b}\times \ln\frac{\varepsilon }{a\times b},$$

Only when *q* satisfies Equation (17), the communication robustness of the hypernetwork will no longer improve. Let θ denote the hyperedge addition threshold and define as follows:

(18) $$\theta =-\frac{1}{b}\times \ln\frac{\varepsilon }{a\times b},$$

By transforming Equation(18), we obtain the following equation:

(19) $$\theta =\frac{1}{b}\times \ln\frac{a\times b}{\varepsilon }.$$

where θ>0. The parameter ε, serving as a small constant, is commonly introduced to avoid numerical instability or overflow in computations. In this study, ε is chosen from the range 0.1 to 0.0001. Using the expression for θ, the hyperedge addition threshold corresponding to each network type and optimization method can be determined. The thresholds for non-uniform hypernetworks are listed in Table 4, while those for uniform hypernetworks are provided in Table A2 in the Appendix A.

According to the experimental data, the hyperedge addition thresholds of some hypernetworks are below 20%. To prevent redundancy in the number of newly added hyperedges and to reduce computational complexity, we uniformly adopt 20% of the initial total number of hyperedges (*M*) as the hyperedge addition ratio in subsequent studies. In contrast, for ER and NW hypernetworks, the estimated thresholds exceed 100%, which is primarily due to the poor performance of the optimization methods proposed in this paper under specific attack scenarios and hypernetwork types, requiring the addition of a large number of hyperedges for the communication robustness to reach saturation. This observation further indicates that the network structure plays a crucial role in determining the performance of the four optimization methods. In the following section, we further investigate the effects of hypernetwork scale and network type on the effectiveness of the proposed optimization methods.

### 4.3. Impact of Hypernetwork Scale on Communication Robustness

**Figure 7:**
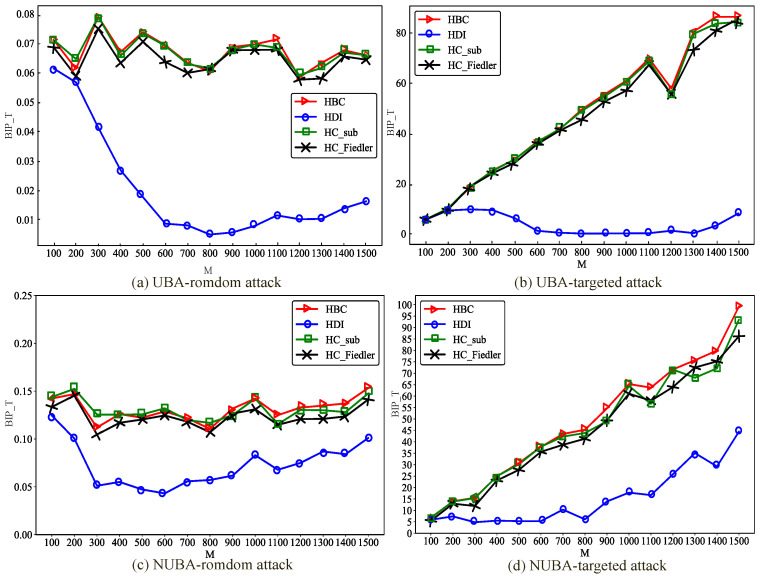
Changes in the best improved performance in uniform and non-uniform BA hypernetworks of different scales. Under the condition of adding the same proportion (0.2×M) of hyperedges, compare the changes of the optimal improvement performance BIP_T with network size *M* for the uniform BA hypernetwork (UBA) and the non-uniform BA hypernetwork (NUBA) under random attacks and targeted attacks.

Figure 7 illustrates the variation of the best improvement performance achieved by the four optimization metrics with respect to the scale of BA hypernetworks, after adding 0.2×M hyperedges using the proposed hypernetwork communication robustness optimization methods under random attacks.

As shown in Figure 7, with the increase in network scale *M*, the best improvement performance exhibits notable differences under different network structures and attack scenarios. Under random attacks, increasing the network scale leads to a continuous enhancement in communication robustness improvement; however, the growth rate remains relatively moderate and gradually stabilizes as the network size becomes large. In contrast, under targeted attacks, the influence of network scale on the best improvement performance is more pronounced. As *M* increases, the performance gains achieved by all optimization methods become more substantial, and the performance disparities among different methods are further amplified. These results indicate that network scale plays a critical role in determining the effectiveness of communication robustness optimization under different attack strategies, with a particularly significant impact observed in targeted attack scenarios.

Moreover, significant differences in improvement performance are observed between random and targeted attacks. In random attacks, nodes are selected uniformly at random, and the attacked nodes may have a limited impact on the global network structure. By contrast, targeted attacks deliberately focus on nodes with large hyperdegrees. In BA hypernetworks, most nodes maintain connectivity by linking to high-hyperdegree nodes, thereby avoiding isolation. Without optimization, targeted attacks can cause BA hypernetworks to rapidly fragment into numerous isolated nodes. The proposed optimization methods mitigate this vulnerability by preemptively adding new hyperedges among such nodes, strengthening their interconnections and effectively preventing network fragmentation. This optimization strategy plays a crucial role in enhancing the communication robustness of hypernetworks.

**Figure 8:**
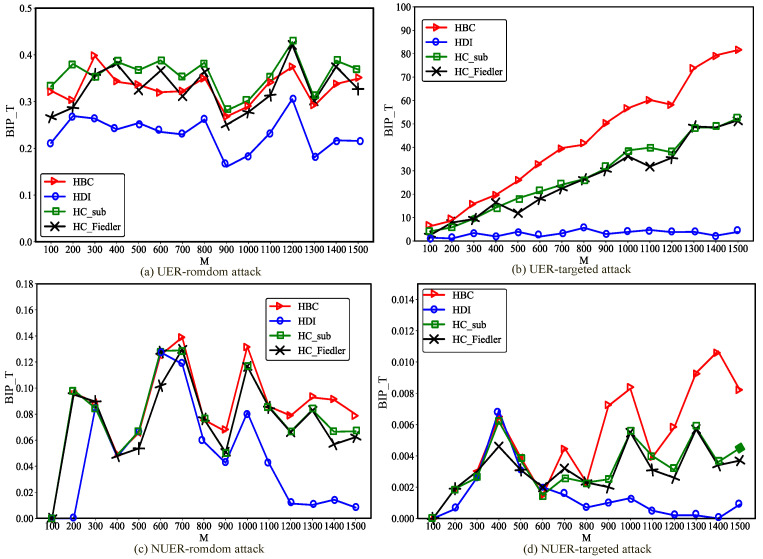
Changes in the best improved performance in uniform and non-uniform ER hypernetworks of different scales. Under the condition of adding the same proportion (0.2×M) of hyperedges, compare the changes of the optimal improvement performance BIP_T with network size *M* for the uniform ER hypernetwork (UER) and the non-uniform ER hypernetwork (NUER) under random attacks and targeted attacks.

Figure 8 illustrates the impact of hypernetwork scale on the improvement of communication robustness in ER hypernetworks. As shown in the figure, as the network scale *M* increases, the communication robustness improvement achieved by all optimization methods exhibits pronounced variation patterns. In uniform ER hypernetworks, enlarging the network scale leads to a gradual enhancement of communication robustness, which eventually stabilizes at larger scales. This observation indicates that, under these attack scenarios, increasing network size contributes positively to overall network robustness. In contrast, in non-uniform ER hypernetworks, the impact of network scale on performance is more complex. Although the overall trend remains upward, significant fluctuations are observed within certain scale intervals. This suggests that communication robustness enhancement in ER hypernetworks is highly sensitive to network scale, resulting in heterogeneous performance across different sizes. Generally, larger-scale networks tend to exhibit more pronounced optimization effects, particularly under targeted attacks, where increasing the network scale plays a crucial role in improving communication robustness. Moreover, for large-scale non-uniform ER hypernetworks facing targeted attacks, the optimization method integrating the HDI metric yields an improvement in hypernetwork communication robustness close to zero. This is because the HDI metric in large-scale hypernetworks is not accurate in judging node positions, as can also be observed in the example provided in Section 2.3.

**Figure 9:**
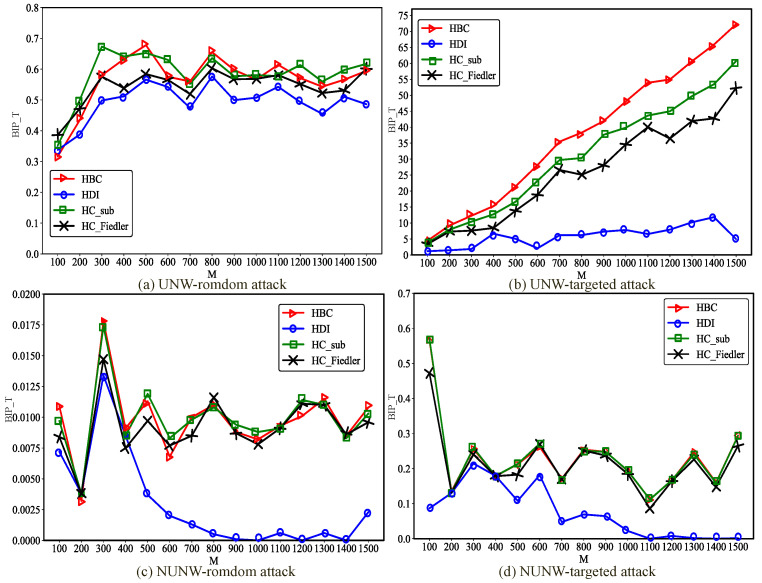
Changes in the best improved performance in uniform and non-Uniform NW hypernetworks of different scales. Under the condition of adding the same proportion (0.2×M) of hyperedges, compare the changes of the optimal improvement performance BIP_T with network size *M* for the uniform NW hypernetwork (UNW) and the non-uniform NW hypernetwork (NUNW) under random attacks and targeted attacks.

As shown in Figure 9, in NW hypernetworks, the influence of network scale on the best improvement performance is largely consistent with that observed in ER hypernetworks. By jointly analyzing the experimental results of ER and NW hypernetworks, it can be observed that, consistent with the findings for BA hypernetworks, targeted attacks yield substantially better optimization performance than random attacks under the same degree of uniformity. This phenomenon can be attributed to similar underlying mechanisms as in BA hypernetworks. In non-uniform NW hypernetworks, the best improvement performance of the optimization method integrating the HDI metric is close to zero, which is consistent with the reason in non-uniform ER hypernetworks. However, an additional distinctive observation emerges: under targeted attack scenarios, uniform hypernetworks consistently outperform non-uniform hypernetworks. In the uniform hypernetworks constructed in this study, all hyperedges are 3-uniform, meaning that each newly added hyperedge connects exactly three nodes. In contrast, in non-uniform hypernetworks, each newly added hyperedge contains a number of nodes equal to the average hyperedge size of the network, which is greater than three. Consequently, non-uniform hypernetworks are more likely to significantly increase the hyperdegrees of peripheral nodes, thereby transforming them into high-hyperdegree nodes. Under targeted attacks, such nodes are more likely to be selected as attack targets, which in turn weakens both the communication robustness and the effectiveness of the optimization.

### 4.4. Impact of Different Hypernetwork Types on Communication Robustness

**Figure A2:**
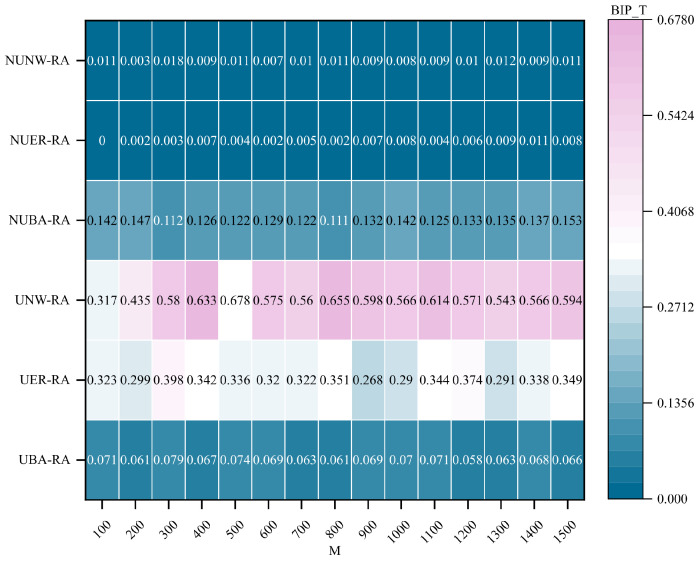
Comparison of optimal improvement performance of communication robustness optimization method integrating HBC indicators under random attacks. Under the random attack (RA) condition, the best improvement performance (BIP_T) of the communication robustness optimization method integrating HBC metrics is compared across different hypernetwork structures. The horizontal axis represents the hypernetwork scale *M*, the vertical axis represents different types of hypernetwork structures, and the shading indicates the BIP_T value.

**Figure A3:**
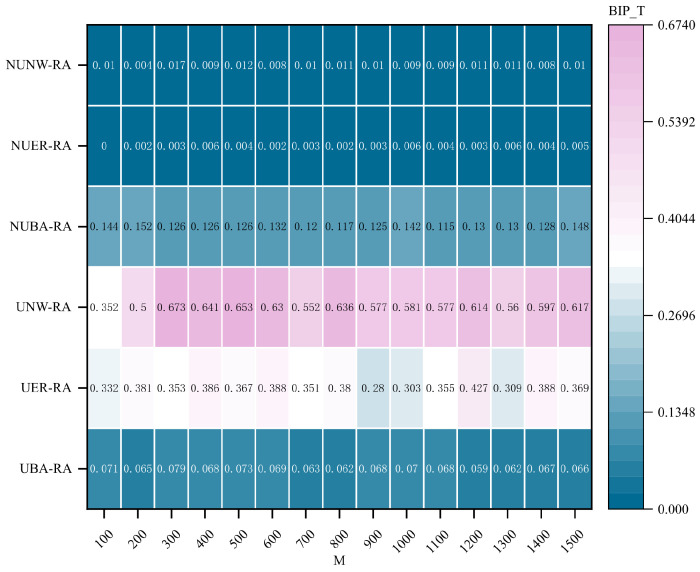
Comparison of optimal improvement performance of communication robustness optimization method integrating HC_sub indicators under random attacks. Under the random attack (RA) condition, the best improvement performance (BIP_T) of the communication robustness optimization method integrating HC_sub metrics is compared across different hypernetwork structures. The horizontal axis represents the hypernetwork scale *M*, the vertical axis represents different types of hypernetwork structures, and the shading indicates the BIP_T value.

**Figure A4:**
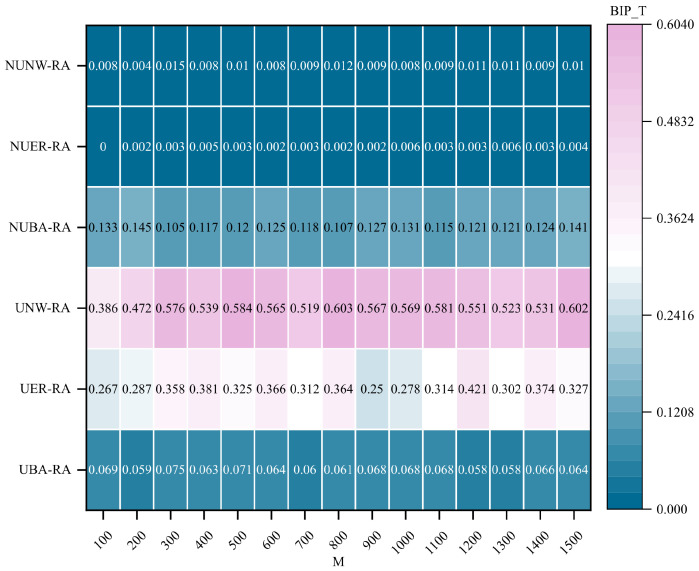
Comparison of optimal improvement performance of communication robustness optimization method integrating HC_Fiedler indicators under random attacks. Under the random attack (RA) condition, the best improvement performance (BIP_T) of the communication robustness optimization method integrating HC_Fiedler metrics is compared across different hypernetwork structures. The horizontal axis represents the hypernetwork scale *M*, the vertical axis represents different types of hypernetwork structures, and the shading indicates the BIP_T value.

**Figure A5:**
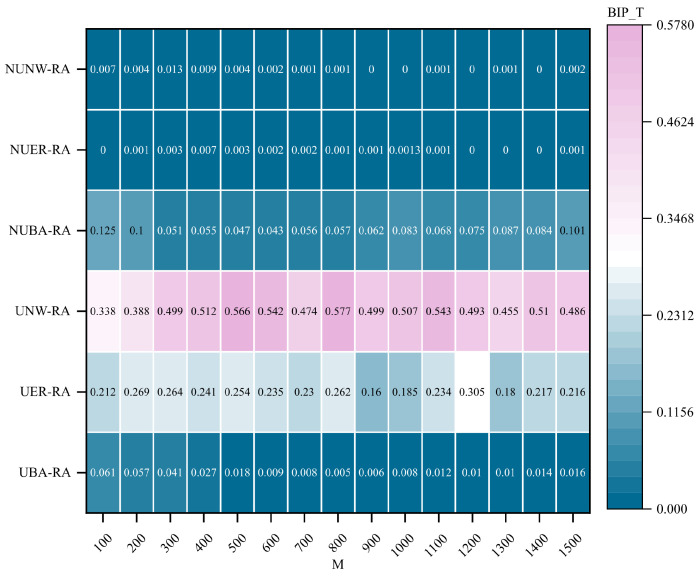
Comparison of optimal improvement performance of communication robustness optimization method integrating HDI indicators under random attacks. Under the random attack (RA) condition, the best improvement performance (BIP_T) of the communication robustness optimization method integrating HDI metrics is compared across different hypernetwork structures. The horizontal axis represents the hypernetwork scale *M*, the vertical axis represents different types of hypernetwork structures, and the shading indicates the BIP_T value.

In this section, we investigate the performance of the same optimization method across different network types. Considering that attacks encountered by real-world complex systems are often intentional, targeted attacks better reflect realistic vulnerability patterns and defense requirements than random attacks. Therefore, the following analysis focuses primarily on the experimental results obtained under targeted attack scenarios. The performance of all methods under random attacks across different networks is presented in Figure A2, Figure A3, Figure A4 and Figure A5 in the Appendix A.

**Figure 10:**
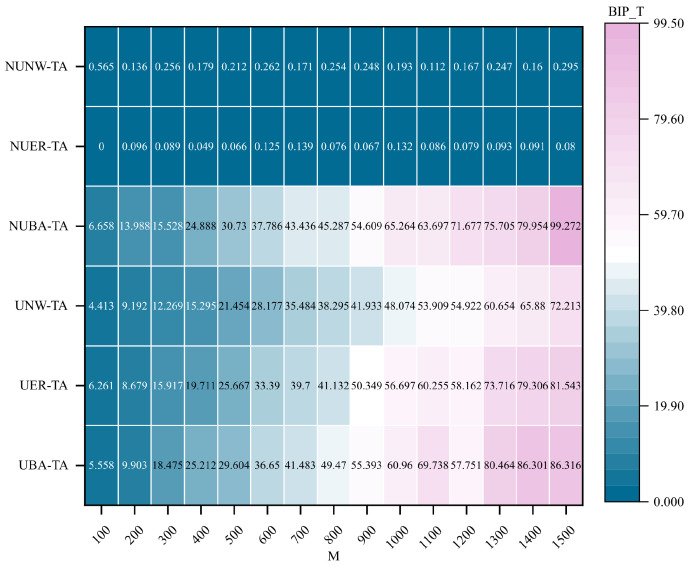
Comparison of optimal improvement performance of communication robustness optimization method integrating HBC indicators under targeted attacks. Under the targeted attack (TA) condition, the best improvement performance (BIP_T) of the communication robustness optimization method integrating HBC metrics is compared across different hypernetwork structures. The horizontal axis represents the hypernetwork scale *M*, the vertical axis represents different types of hypernetwork structures, and the shading indicates the BIP_T value.

As indicated by the experimental results in Figure 10, the communication robustness optimization method incorporating the HBC metric exhibits significant performance disparities across different network structures. Among them, BA hypernetworks demonstrate strong adaptability. Specifically, the non-uniform BA hypernetwork achieves the highest optimization performance, reaching approximately 99.272, which significantly outperforms all other network types. The uniform BA hypernetwork also shows excellent performance, with a final optimization value of 86.316. In contrast, the optimization effects observed in ER and NW networks are generally weaker. The peak improvement in the non-uniform ER network (NUER-TA) is only 0.139, while that in the uniform ER network (UER-TA) reaches 81.543. For NW networks, the non-uniform NW network (NUNW-TA) and the uniform NW network (UNW-TA) achieve peak values of merely 0.565 and 72.213, respectively. These results indicate that the proposed optimization method exhibits a pronounced advantage in BA hypernetwork topologies with scale-free characteristics.

**Figure 11:**
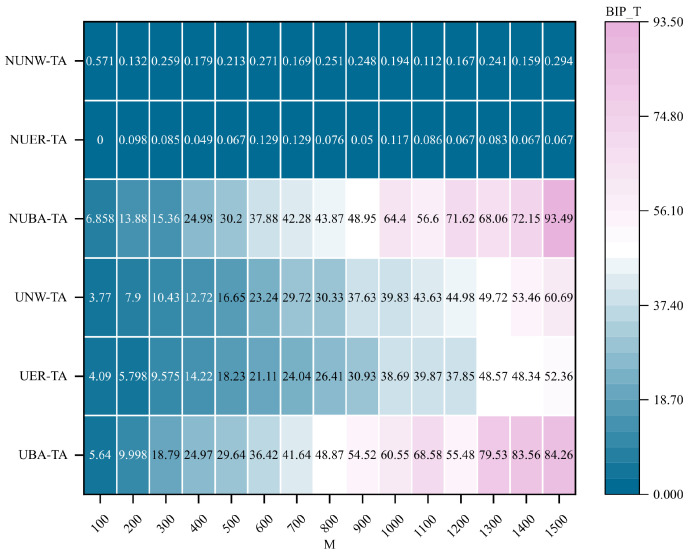
Comparison of optimal improvement performance of communication robustness optimization method integrating HC_sub indicators under targeted attacks. Under the targeted attack (TA) condition, the best improvement performance (BIP_T) of the communication robustness optimization method integrating HC_sub metrics is compared across different hypernetwork structures. The horizontal axis represents the hypernetwork scale *M*, the vertical axis represents different types of hypernetwork structures, and the shading indicates the BIP_T value.

**Figure 12:**
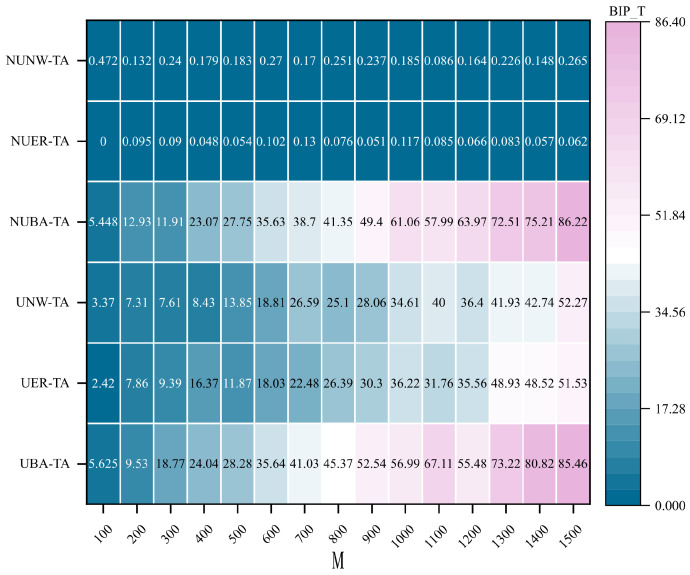
Comparison of optimal improvement performance of communication robustness optimization method integrating HC_Fiedler indicators under targeted attacks. Under the targeted attack (TA) condition, the best improvement performance (BIP_T) of the communication robustness optimization method integrating HC_Fiedler metrics is compared across different hypernetwork structures. The horizontal axis represents the hypernetwork scale *M*, the vertical axis represents different types of hypernetwork structures, and the shading indicates the BIP_T value.

The experimental results shown in Figure 11 and Figure 12 further reveal that the communication robustness optimization methods incorporating the HC_Fiedler and HC_sub metrics exhibit a clear performance hierarchy across different network structures, consistent with the observations in Figure 10. BA hypernetworks again demonstrate substantial superiority, with the non-uniform BA hypernetwork achieving the highest optimization performance, markedly exceeding that of other network types. In contrast, the performance improvements in ER and NW networks remain relatively limited.

**Figure 13:**
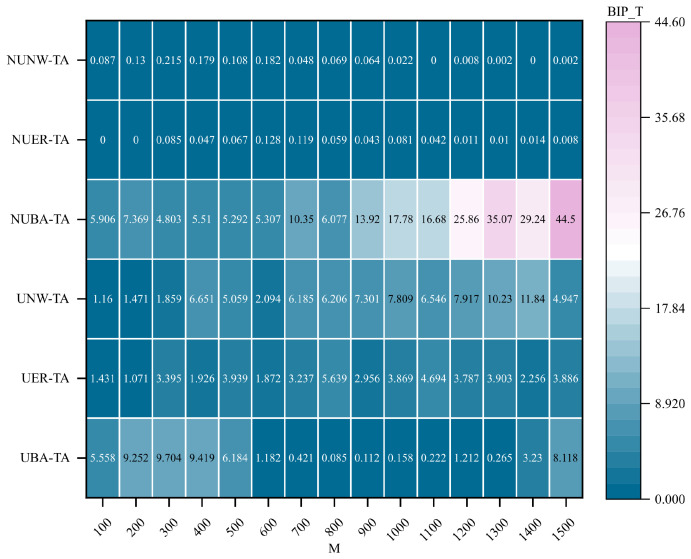
Comparison of optimal improvement performance of communication robustness optimization method integrating HDI indicators under targeted attacks. Under the targeted attack (TA) condition, the best improvement performance (BIP_T) of the communication robustness optimization method integrating HDI metrics is compared across different hypernetwork structures. The horizontal axis represents the hypernetwork scale *M*, the vertical axis represents different types of hypernetwork structures, and the shading indicates the BIP_T value.

As shown by the experimental results in Figure 13, the communication robustness optimization method based on the HDI metric exhibits extremely limited performance in non-uniform NW hypernetworks. All recorded values remain at low levels, with a peak of only 0.215, followed by a rapid decay toward values close to zero (e.g., 0.002, 0.008, and multiple zero entries) as the process progresses. This performance is significantly inferior to that observed in previously analyzed network structures, indicating that under the given experimental conditions, this method has weak adaptability to non-uniform NW topologies and fails to achieve practically meaningful optimization. The sharp decay trend further suggests that this method is particularly prone to failure in NW-type networks when network parameters or structural characteristics vary. Nevertheless, the HDI-based optimization method still achieves its best performance in non-uniform BA hypernetworks.

### 4.5. WIKI-VOTE Social Hypernetwork Communication Robustness Optimization

The WIKI-VOTE social network dataset used in this study is obtained from the Network Repository website. After processing and cleaning the dataset, 789 social individuals and 545 social group relationships formed between these individuals were extracted from the WIKI-VOTE social hypernetwork. From the original data, it was observed that a social individual can correspond to multiple social group relationships, and the number of social individuals in each group relationship varies, which aligns with the characteristics of a non-uniform structure. Therefore, the WIKI-VOTE social hypernetwork is suitable for constructing a non-uniform hypernetwork. In this paper, the 789 social individuals are abstracted as nodes in the hypernetwork, and the 545 social group relationships are abstracted as hyperedges in the hypernetwork.

The hypernetwork communication robustness optimization method was applied to the WIKI-VOTE social hypernetwork, and the improvement in the BIP_T of the network’s communication robustness under random and targeted attacks was obtained. To ensure the validity of the experiment, the results were averaged over 50 trials.

**Figure 14:**
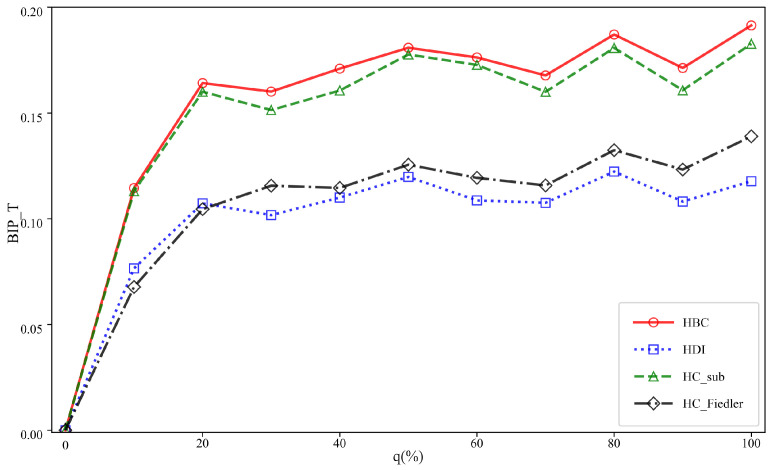
Trend of the best improvement performance of the WIKI-VOTE social hypernetwork under random attacks as the number of added hyperedges increases.

As shown in Figure 14, all four hypernetwork connectivity robustness optimization methods can enhance the communication robustness of the social hypernetwork. Among them, the final value of HBC is significantly higher; HC_sub outperforms HC_Fiedler in both growth rate and final value; while HDI still shows the poorest optimization effect.

**Figure 15:**
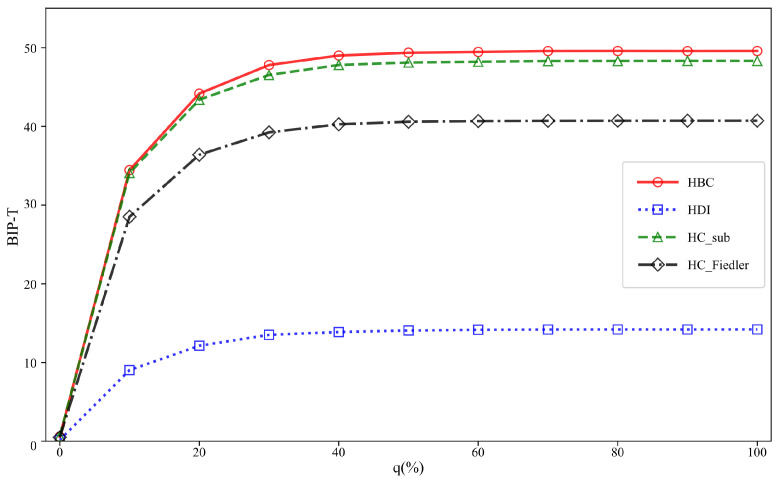
Trend of the best improvement performance of the WIKI-VOTE social hypernetwork under targeted attacks as the number of added hyperedges increases.

As shown in Figure 15, all four hypernetwork connectivity robustness optimization methods can significantly enhance the communication robustness of the social hypernetwork. Among them, HBC and HC_sub show significant optimization effects, with both exhibiting similar growth rates and final values. HC_Fiedler follows, while HDI still shows the poorest optimization effect.

## 5. Conclusions

This paper combines the structural characteristics of hypernetworks with four metrics—hyper-betweenness centrality, hyper-centrality of feature subgraph, hyper-centrality Fiedler centrality, and hyperdistance entropy—along with the communication robustness optimization method to enhance the communication robustness of hypernetworks. The purpose of these metrics is to improve the communication robustness of the hypernetwork, and their effectiveness was verified through simulation experiments conducted on both synthetic and empirical hypernetworks. The following conclusions can be drawn from the experimental results:

The hypernetwork communication robustness optimization method integrating four metrics proposed in this study can effectively improve the communication robustness in three typical hypernetwork structures (Barabási–Albert, Erdos–Renyi and Newman–Watts) and demonstrates significant optimization effects in the real-world social hypernetwork WIKI-VOTE. In terms of the performance of each metric-based method, the approach based on hyper-betweenness centrality (HBC) performs best across all networks, followed by the methods based on hyper-centrality of feature subgraph (HC_sub) and Fiedler centrality (HC_Fiedler), while the method based on hyperdistance entropy (HDI) performs poorly in all tested networks. In addition, the research systematically explores the influence of the hyperedge addition ratio *q*, hypernetwork scale, and network type on the optimization effect, and theoretically derives the corresponding hyperedge addition threshold θ. Further experiments show that the larger the network scale, the stronger the practicability of this fusion method; in most cases, the optimization effect of communication robustness in Barabási–Albert (BA) hypernetworks is more prominent under targeted attacks; while under random attacks, the experimental performance of Erdos–Renyi (ER) and Newman–Watts (NW) hypernetworks is slightly better than that of BA hypernetworks.

This study provides methods and references for optimizing the communication robustness of hypernetworks. Future research will continue to explore the application of a hypernetwork communication robustness optimization approach that integrates four metrics—hyper-betweenness centrality, hyper-centrality of feature subgraphs, the Fiedler value, and hyperdistance entropy—in other higher-order networks. It will also delve into how hypernetwork structure influences the effectiveness of such optimization methods. Furthermore, we will investigate ways to combine these methods with neural networks to enhance hypernetwork robustness. Additionally, future work may further refine hypernetwork robustness optimization from the perspective of algebraic spectral theory, such as by utilizing the Laplacian operator. All of these directions represent valuable avenues for subsequent research. Such studies will further improve the robustness of higher-order networks and offer novel insights for their optimization.

## Figures and Tables

**Table 1:** Ranking of nodes in hypernetwork *H* based on the four centrality metrics.

Rank	HBC	HC_sub	HC_Fiedler	HDI
1	$v_{2}$	$v_{2}$	$v_{7}$	$v_{6}$
2	$v_{0}$	$v_{6}$	$v_{9}$	$v_{0}$
3	$v_{6}$	$v_{7}$	$v_{6}$	$v_{2}$
4	$v_{9}$	$v_{0}$	$v_{0}$	$v_{1}$
5	$v_{7}$	$v_{9}$	$v_{2}$	$v_{9}$
6	$v_{5}$	$v_{5}$	$v_{8}$	$v_{7}$
7	$v_{1}$	$v_{8}$	$v_{1}$	$v_{3}$
8	$v_{3}$	$v_{3}$	$v_{5}$	$v_{4}$
9	$v_{4}$	$v_{4}$	$v_{3}$	$v_{5}$
10	$v_{8}$	$v_{1}$	$v_{4}$	$v_{8}$

**Table 2:** Main parameters and their values for simulation experiments of hypernetwork communication robustness optimization methods.

Parameter Name	Parameter Definition	Value Range
*N*	Total number of nodes	[100, 1200]
*k*	Number of nodes per hyperedge in uniform hypernetwork	3
$|e_{i}|$	Number of nodes per hyperedge in non-uniform hypernetwork	[3,10]
*M*	Total number of hyperedges	[100, 1500]
*q*	Proportion of newly added hyperedges	[0,1]

**Table 3:** Theoretical expressions of robustness optimization curves for non-uniform hypernetworks.

Network Type	Optimization Method	Expression
Non-uniform BA hypernetwork Random attack	HBC	$BIP\_T=0.18(1-e^{-0.09q})$
	HC_sub	$BIP\_T=0.17(1-e^{-0.10q})$
	HC_Fiedler	$BIP\_T=0.16(1-e^{-0.09q})$
	HDI	$BIP\_T=0.12(1-e^{-0.10q})$
Non-uniform BA hypernetwork Targeted attack	HBC	$BIP\_T=110.62(1-e^{-0.10q})$
	HC_sub	$BIP\_T=104.40(1-e^{-0.10q})$
	HC_Fiedler	$BIP\_T=97.44(1-e^{-0.09q})$
	HDI	$BIP\_T=52.74(1-e^{-0.08q})$
Non-uniform ER hypernetwork Random attack	HBC	$BIP\_T=0.01(1-e^{-0.11q})$
	HC_sub	$BIP\_T=0.008(1-e^{-0.12q})$
	HC_Fiedler	$BIP\_T=0.006(1-e^{-0.11q})$
	HDI	$BIP\_T=0(1-e^{-0.23q})$
Non-uniform ER hypernetwork Targeted attack	HBC	$BIP\_T=0.08(1-e^{-0.15q})$
	HC_sub	$BIP\_T=0.07(1-e^{-0.15q})$
	HC_Fiedler	$BIP\_T=0.06(1-e^{-0.15q})$
	HDI	$BIP\_T=0.01(1-e^{-0.14q})$
Non-uniform NW hypernetwork Random attack	HBC	$BIP\_T=0.01(1-e^{-0.10q})$
	HC_sub	$BIP\_T=0.01(1-e^{-0.09q})$
	HC_Fiedler	$BIP\_T=0.01(1-e^{-0.09q})$
	HDI	$BIP\_T=0(1-e^{-0.09q})$
Non-uniform NW hypernetwork Targeted attack	HBC	$BIP\_T=0.31(1-e^{-0.18q})$
	HC_sub	$BIP\_T=0.31(1-e^{-0.18q})$
	HC_Fiedler	$BIP\_T=0.28(1-e^{-0.18q})$
	HDI	$BIP\_T=0.01(1-e^{-0.01q})$

**Table 4:** Load threshold θ(%) of four optimization methods under different non-uniform hypernetwork types and attack modes.

Network Type	Optimization Method	Load Threshold θ(%)
Non-uniform BA hypernetwork Random attack	HBC	56.53
	HC_sub	51.36
	HC_Fiedler	55.22
	HDI	47.87
Non-uniform BA hypernetwork Targeted attack	HBC	47.06
	HC_sub	46.48
	HC_Fiedler	49.71
	HDI	75.56
Non-uniform ER hypernetwork Random attack	HBC	21.79
	HC_sub	18.8
	HC_Fiedler	17.2
	HDI	0
Non-uniform ER hypernetwork Targeted attack	HBC	31.9
	HC_sub	31.0
	HC_Fiedler	30.0
	HDI	18.9
Non-uniform NW hypernetwork Random attack	HBC	23.02
	HC_sub	24.4
	HC_Fiedler	24.4
	HDI	0
Non-uniform NW hypernetwork Targeted attack	HBC	35.1
	HC_sub	35.1
	HC_Fiedler	34.6
	HDI	0

## Data Availability

The datasets generated and analyzed during the current study are available from the first author, Lei Chen, upon reasonable request.

## Appendix A. Supplementary Experiment

**Table A1:** Theoretical expressions of robustness optimization curves for uniform hypernetworks.

Network Type	Optimization Method	Expression
Uniform BA hypernetwork Random attack	HBC	$BIP\_T=0.07(1-e^{-0.13q})$
	HC_sub	$BIP\_T=0.07(1-e^{-0.13q})$
	HC_Fiedler	$BIP\_T=0.07(1-e^{-0.13q})$
	HDI	$BIP\_T=0.02(1-e^{-0.12q})$
Uniform BA hypernetwork Targeted attack	HBC	$BIP\_T=90.20(1-e^{-0.15q})$
	HC_sub	$BIP\_T=88.62(1-e^{-0.14q})$
	HC_Fiedler	$BIP\_T=89.87(1-e^{-0.14q})$
	HDI	$BIP\_T=13.02(1-e^{-0.04q})$
Uniform ER hypernetwork Random attack	HBC	$BIP\_T=0.70(1-e^{-0.03q})$
	HC_sub	$BIP\_T=0.60(1-e^{-0.04q})$
	HC_Fiedler	$BIP\_T=0.57(1-e^{-0.04q})$
	HDI	$BIP\_T=0.36(1-e^{-0.04q})$
Uniform ER hypernetwork Targeted attack	HBC	$BIP\_T=134.26(1-e^{-0.04q})$
	HC_sub	$BIP\_T=91.53(1-e^{-0.04q})$
	HC_Fiedler	$BIP\_T=90.58(1-e^{-0.04q})$
	HDI	$BIP\_T=41.62(1-e^{-0.01q})$
Uniform NW hypernetwork Random attack	HBC	$BIP\_T=1.17(1-e^{-0.04q})$
	HDI	$BIP\_T=0.80(1-e^{-0.05q})$
	HC_sub	$BIP\_T=1.13(1-e^{-0.04q})$
	HC_Fiedler	$BIP\_T=1.07(1-e^{-0.04q})$
Uniform NW hypernetwork Targeted attack	HBC	$BIP\_T=106.25(1-e^{-0.05q})$
	HC_sub	$BIP\_T=95.25(1-e^{-0.05q})$
	HC_Fiedler	$BIP\_T=91.33(1-e^{-0.04q})$
	HDI	$BIP\_T=48.28(1-e^{-0.01q})$

**Table A2:** Load threshold θ(%) of four optimization methods under different uniform hypernetwork types and attack modes.

Network Type	Optimization Method	Load Threshold θ(%)
Uniform BA hypernetwork Random attack	HBC	34.7
	HC_sub	34.7
	HC_Fiedler	34.7
	HDI	26.5
Uniform BA hypernetwork Targeted attack	HBC	32.7
	HC_sub	34.3
	HC_Fiedler	34.5
	HDI	98.8
Uniform ER hypernetwork Random attack	HBC	178.2
	HC_sub	137.0
	HC_Fiedler	135.8
	HDI	124.2
Uniform ER hypernetwork Targeted attack	HBC	157.2
	HC_sub	147.6
	HC_Fiedler	147.3
	HDI	603.1
Uniform NW hypernetwork Random attack	HBC	153.7
	HC_sub	152.8
	HC_Fiedler	151.4
	HDI	119.8
Uniform NW hypernetwork Targeted attack	HBC	125.5
	HC_sub	123.4
	HC_Fiedler	147.5
	HDI	618.0
